# A randomized controlled trial investigating the effect of a diet low in fermentable oligosaccharides, disaccharides, monosaccharides, and polyols on the intestinal microbiome and inflammation in patients with ulcerative colitis: study protocol for a randomized controlled trial

**DOI:** 10.1186/s13063-020-4108-7

**Published:** 2020-02-18

**Authors:** Alireza Milajerdi, Omid Sadeghi, Seyed Davar Siadat, Seyed Ali Keshavarz, Alireza Sima, Homayoon Vahedi, Peyman Adibi, Ahmad Esmaillzadeh

**Affiliations:** 1 Students Scientific Research Center, Tehran University of Medical Sciences, Tehran, Iran; 2 Department of Community Nutrition, School of Nutritional Sciences and Dietetics, Tehran University of Medical Sciences, PO Box 81745, Tehran, Iran; 3 Department of Mycobacteriology and Pulmonary Research, Microbiology Research Center, Pasteur Institute of Iran, Tehran, Iran; 4 Department of Clinical Nutrition, School of Nutritional Sciences and Dietetics, Tehran University of Medical Sciences, Tehran, Iran; 5 Digestive Disease Research Institute, Shariati Hospital, Tehran University of Medical Science, Tehran, Iran; 6 Integrative Functional Gastroenterology Research Center, Isfahan University of Medical Sciences, Isfahan, Iran; 7 Obesity and Eating Habits Research Center, Endocrinology and Metabolism Molecular Cellular Sciences Institute, Tehran University of Medical Sciences, Tehran, Iran; 8 Food Security Research Center, Department of Community Nutrition, School of Nutrition and Food Science, Isfahan University of Medical Sciences, Isfahan, Iran

**Keywords:** FODMAP diet, Inflammation, Microbiota, RCT, Ulcerative colitis

## Abstract

**Background:**

No conclusive treatment is available for irritable bowel disease (IBD). Adherence to a diet low in fermentable oligosaccharides, disaccharides, monosaccharides, and polyols (FODMAPs) might alleviate clinical symptoms of IBD. However, no study has investigated the effect of low FODMAPs diet on the intestinal microbiota and inflammatory biomarkers in patients with IBD. The aim of current study is to examine the effect a low FODMAP diet on IBD symptoms, inflammation, and the intestinal microbiota in patients with ulcerative colitis.

**Methods and analysis:**

This study is a randomized clinical trial. Thirty patients with mild to moderate ulcerative colitis will be randomly allocated to receive a low FODMAP diet (*n* = 15) or to continue their usual diet as control (*n* = 15), for 4 weeks. The quantity of intestinal microbiota including Clostridium cluster IV, *Faecalibacterium prausnitzii*, Rosburia spp., Lactobacillus spp., Bifidobacteria spp., *Akkermansia muciniphila*, *Bacteroides fragilis*, and Ruminococcus spp., and the *Firmicutes* to *Bacteroidetes* ratio and calprotectin and lactoferrin levels will be explored in fecal samples from patients. In addition, anthropometric measures and biochemical assessments including serum concentrations of highly sensitive-C reactive protein (hs-CRP), tumour necrosis factor-α (TNF-α) and IL-1β will be taken from patients at baseline and end of the study. The study has been registered in IRCT (IRCT20181126041763N1; registration date: 2019-01-18).

**Discussion:**

Consumption of a low-FODMAP diet might decrease systemic and intestinal inflammation, change the bacterial population in the gut, and modulate clinical symptoms in patients with ulcerative colitis. Further studies investigating the effect of such a diet on other variables, including other bacterial species and inflammatory cytokines, are required to confirm future findings of this trial.

## Background

Inflammatory bowel disease (IBD), a chronic gastrointestinal disease with frequent episodes of diarrhea and constipation, usually occurs in adults aged 20–40 years [1]. It imposes a great burden on the health system and personal life [2]. Although not life-threatening, it causes several disabilities, which may continue throughout life [3].

The exact etiology of IBD is still not understood; however, disturbances in the immune system, inflammation, and genetic predisposing factors are among the most identified risk factors [4]. Gut bacteria are suggested to play a role in this regard [5, 6]. Immune dysfunction may damage the natural microflora in the intestine and consequently accelerate IBD symptoms [7]. In addition, changes in the gut microbiota may cause harmful immunological responses in the intestinal mucosa [7, 8].

No conclusive treatment is available for IBD. Restriction of fermentable oligosaccharides, disaccharides, monosaccharides, and polyols (FODMAPs) in some gastrointestinal (GI) problems has been resulted in symptoms alleviation [9, 10]. However, it should be noted that FODMAPs have prebiotic properties in humans [11]. Therefore, a low FODMAP diet might result in reduced growth of beneficial bacteria in the intestine. In addition, fermentation of FODMAPs by the gut microbiota produces a considerable amount of short-chain fatty acids (SCFA), such as butyrate, which could be used as a fuel for intestinal cells [12]. Therefore, although reduction in FODMAPs may alleviate IBD symptoms partially due to reduction in bacterial fermentation [13], the exact mechanism through which a low FODMAPs diet may influence these symptoms are unknown. There is no study available investigating the effect of a low FODMAPs diet on the intestinal microbiota and inflammatory biomarkers in patients with IBD. Therefore, this study aimed to examine the effect of consumption of a low FODMAP diet on IBD symptoms, inflammation and gut microbiota in patients with ulcerative colitis (UC).

## Methods

### Participants

**Fig. 1:**
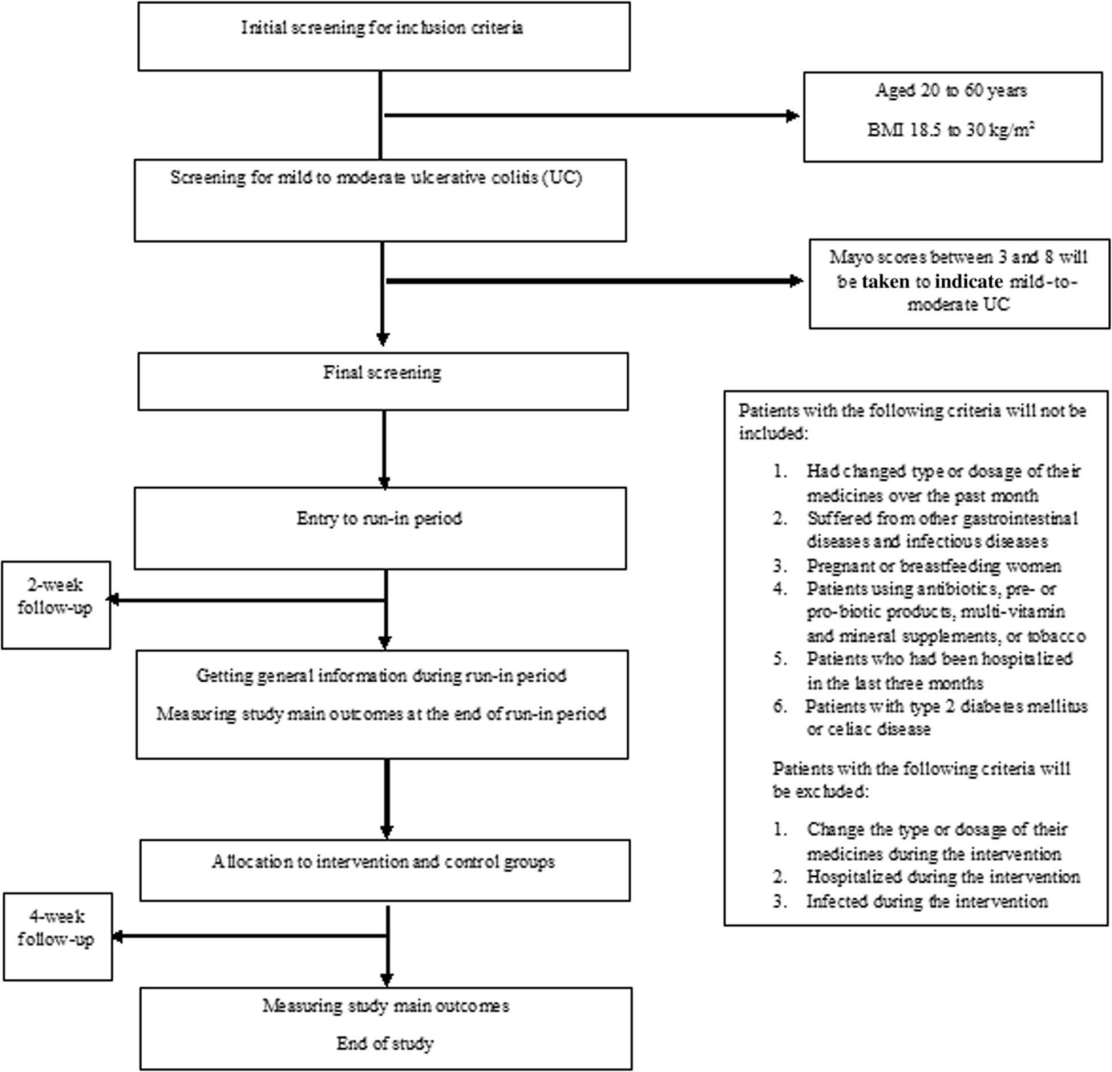
Flow diagram of study selection. BMI, body mass index

**Fig. 2:**
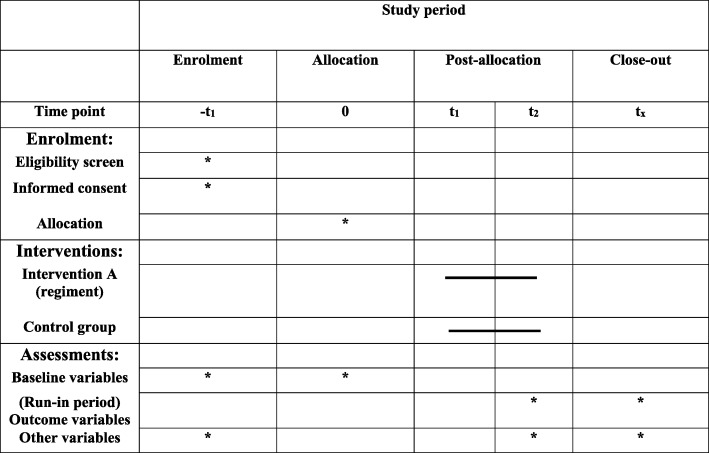
Flow diagram of the study process

This study is a randomized clinical trial. Participants will be recruited from Shariati and Imam Khomeini hospitals, Tehran, Iran. Subjects (aged 20–60 years, body mass index (BMI) 18.5–30 kg/m^2^) with mild to moderate UC who are in disease remission with stable drug therapy will be selected for this study. UC will be diagnosed by a gastroenterologist using the Mayo score. Scores of 3–8 will be considered as mild to moderate UC [14]. Patients who had changed type or dosage of their medicines over the past month, suffered from other gastrointestinal diseases including cancer and infectious diseases, who are pregnant or breastfeeding, or are using antibiotics, prebiotic or probiotic products, multi-vitamin, and mineral supplements, or tobacco will not be included. We will not include patients who had been hospitalized in the last 3 months or patients with type 2 diabetes mellitus or celiac disease. Individuals who change the type or dosage of their medicines during the intervention, or are hospitalized or develop infection will be excluded. All participants will provide informed written consent. Moreover, the study protocol has been recorded in the irct.ir website (IRCT20181126041763N1; registration date: 2019-01-18). The flow-diagram of the project is shown in Fig. 1. Flow-diagram of study process is shown in Fig 2 (Additional file 1).

### Study design

The study was designed according to the Standard Protocol Items: Recommendations for Interventional Trials (SPIRIT) 2013 checklist. After recruitment, participants will enter into a 2-week run-in period, during which general information on diet, physical activity, type and dosage of medications will be obtained. All measurements, including anthropometric and biochemical assessments, will be made at this stage, and then participants will be stratified based on age (20–40 and 40–60 years), gender (male/female), body mass index (18.5–24.9 and 25–30), and type of medicines (anti-inflammatory and immunosuppressant) into different strata. Actually, first we will enroll a participant who meets the inclusion criteria in a stratum. Then, based on these variables the second person, who is matched with the first one in that stratum would be gone to the same stratum. So, we would have two similar persons (in terms of the aforementioned variables) in a stratum. Then, the two persons in a single stratum would be randomly assigned into the intervention and non-intervention groups. Therefore, we would have 15 strata (each consisting of two similar persons) in total and then individuals in the strata would be randomly allocated to the aforementioned groups. So, we would have 15 people in each group and the groups would be almost similar in terms of these variables. A person who is not part of the study will randomly allocate participants. Participants in the intervention group will receive a low-FODMAP diet according to their usual dietary intakes, whereas participants in the control group will receive their usual diet without any modification, for 4 weeks. Participants in both groups will receive the usual dietary recommendations for patients with IBD. To evaluate participants’ compliance, three 24-h dietary recalls will be taken throughout the intervention. Participants will be asked not to change their life-style and medicines during the study. Again, all measurements will be examined at the end of trial.

### Patient and public involvement

Patients and the public were not involved in the development of this study.

### Intervention

A 2-week dietary menu will be provided for each participant. First, we will calculate the basic metabolic rate (BMR) of each subject according to the participant’s age, sex, height and current weight, using the Harris-Benedict formula. Then, daily energy requirements of each participant will be estimated based on participants’ physical activity and the thermogenic effect of foods (TEF). Based on the total energy requirement of each individual, we will design a diet consisting of 55% of total energy from carbohydrates, 25% from fats, and 20% from proteins. Then a menu cycle will be written considering the foods high in FODMAP, which will be restricted in these menu cycles. All dietary menus would be designed to include six daily meals, including three main meals and three snacks.

### Assessment of study outcomes

#### Primary outcomes

The main primary outcomes in the current study would be fecal calprotectin and lactoferrin as well as gut microbiota including Clostridium cluster IV, *Faecalibacterium prausnitzii*, Rosburia spp., Lactobacillus spp., Bifidobacteria spp., *Akkermansia muciniphila*, *Bacteroides fragilis*, and Ruminococcus spp. and the *Firmicutes* to *Bacteroidete*s ratio.

To examine gut microbiota and fecal calprotectin and lactoferrin, stool samples (10 g) from one defecation will be taken from each individual at study baseline and end of trial. The samples will be immediately stored at − 20 C° until further analysis. Bacterial DNA will be extracted using the QIAamp (QIAGEN) commercial fecal DNA kit. Calprotectin is a calcium binding protein commonly derived from neutrophils and monocytes and in to a lesser extent from macrophages [15]. Calprotectin is found in stool and plasma, and its concentration increases throughout inflammatory conditions including IBD, and infection [15, 16]. The quantity of intestinal microbiota including Clostridium cluster IV, *F. prausnitzii*, Rosburia spp., Lactobacillus spp., Bifidobacteria spp., *A. muciniphila*, *B. fragilis*, and Ruminococcus spp., as well as the *Firmicutes* to *Bacteroidetes* ratio, will be determined using commercial real-time PCR kits (QIAamp, QIAGEN). In addition, fecal concentrations of calprotectin and lactoferrim will also be measured using an enzyme-linked immunosorbent assay. Therefore, our primary outcomes will be the mean difference in changes from baseline to 4-week follow up in calprotectin concentrations and quantity of gut microbiota between the intervention and control groups.

#### Secondary outcomes

The main secondary outcome variables in the current study will be serum concentrations of highly sensitive-C reactive protein (hs-CRP), tumour necrosis factor-α (TNF-α) and IL-1β. After 12 h of fasting, a 10-cc blood sample will be taken from each patient at study baseline and end of trial. Total cell blood counts (CBC), hemoglobin and hematocrit concentrations will be measured by a cell counter. Erythrocyte sedimentation rate (ESR) will be measured using the Wintergreen method. Then, serum will be isolated from the whole blood and stored at − 80 C^0^. Serum levels of inflammatory cytokines, including hs-CRP, TNF-α, and IL-1β will be measured by a commercial ELISA kit. CRP is produced almost exclusively in liver and by hepatocytes in response to pro-inflammatory cytokines such as IL-6, TNF-α, and IL-1. It is a reactive acute-phase protein with a short half-life [17]. A significant association has been found between the severity of IBD [18] and hs-CRP, TNF-α, and IL-1β. Therefore, our secondary outcomes will be the mean difference between the intervention and control groups in changes in hs-CRP, TNF-α, and IL-1β from baseline to 4-week follow up.

### Assessment of other variables

Data on dietary habits and dietary intakes of participants will be obtained using standard questionnaires in the run-in period. In addition, demographic data, and medical history will be examined using a general information questionnaire during this period. Moreover, participants will be asked to provide three 24-h food recalls throughout this period.

Anthropometric measures including weight, height, and waist circumference (WC) will be measured and BMI (weight (kilograms) divided by the squared height (meters)) will be calculated at the study beginning and end. Participants will be weighed with minimum clothing and without shoes, to the nearest 100 g using a digital scale. Standing height will be measured without shoes, to the nearest 0.5 cm using a standard stadiometer. WC will be measured using a strip meter at the mid-distance interval between the super elliptic bone and the last rib, to the nearest 0.5 cm. In addition, blood pressure will be measured twice at the right arm using a mercury barometer calibrated by the Institute of Standardization and Industrial Research, with a 15- min interval in between measurements, while the patient is sitting quietly for 5 min. The average of two measurements will be analyzed for the participants’ systolic and diastolic blood pressure. Moreover, participants’ physical activity will also be estimated at beginning and end of the study, using 3-day physical activity records.

Participants’ sublingual body temperature will be measured twice with a 15-min interval between measurements, using a standard mercuric thermometer in a room with a temperature of 24–26 °C. The mean of the two measurements will be analyzed as the participant’s body temperature. The mean of two manual counts of pulse rate from the left wrist within 1 min (with a 5-min interval between counts) will be analyzed as the pulse number per minute. In addition, the severity of IBD will be examined using the 9-point partial Mayo score [14], participants’ quality of life assessed by the IBDQ-9 self-administrated questionnaire [19], disabilities due to IBD assessed by the IBD Disability Index (IBD-DI) questionnaire [20], and depression and anxiety assessed by the HADS Hospital Scale [21]. In addition, participants’ psychological stress will also be examined using the General Health Questionnaire (GHQ) [22].

### Study sample size calculation

By considering a type I error of 5% (α = 0.05) and type II error of 20% (β = 0.20, power = 80%) and the primary outcome variable of expected difference between the intervention and control groups in mean fecal calprotectin, calculated based on the two sided *t* test, we calculated the required sample size using the following formula:

$$ n=\frac{{\left({\mathrm{Z}}_{1-\frac{\alpha }{2}}+{\mathrm{Z}}_{1-\beta}\right)}^2\times \left({S_1}^2+{S_2}^2\ \right)}{\left({\upmu_1}^2-{\upmu_2}^2\right)} $$

(S1= 25.88, S2= 21.99, μ1= 26.6, μ2= 4)

Based on this formula and allowing for 10% drop-out in each group, we will need a sample size of 15 persons for each group.

### Statistical analysis

Normality of data will be examined using the Kolmogorov-Smirnov test. Continuous variables will be compared between the two groups by the independent sample *t* test, whereas the chi-square test will be used to compare categorical variables. Normally distributed continuous variables will be reported as mean ± SD, and as median and IQR if they are not normally distributed; categorical data will be reported as absolute and relative percentage frequencies. The effect of the study intervention on desirable outcomes will be measured using the independent sample *t* test for normally distributed data and the Wilcoxon test for non-normally distributed data. We will adjust our final results for potential confounding factors using multivariable regression modeling. Statistical analyses will be done using SPSS, version 21. *P* values <0.05 will be considered statistically significant.

## Discussion

No conclusive treatment is available for IBD. Lack of conclusive treatment for IBD and various complications associated with medicines have drawn attention to the use of alternative medicines [23]. Adherence to an appropriate diet is among the recently investigated approaches to stop IBD progression [24]. Consumption of a low-FODMAP diet decreases the fermentation rate of food remnants in the gut [10]. This will subsequently decrease bacterial growth and probably the associated inflammation, which is the core player in IBD incidence and progression [9, 25]. However, it is not clear how this process changes the intestinal microbiota. In addition, along with the probable reduction in inflammation, diminished fermentation by intestinal bacteria reduces fluctuant and may also reduce clinical symptoms of IBD [9]. To the best of our knowledge, no previous studies have investigated the effect of a low FODMAPs diet on the intestinal microbiota and inflammatory biomarkers in patients with IBD.

### Strengths

This is the first clinical trial investigating the effect of a low-FODMAP diet on the intestinal microbiota and inflammation in patients with IBD. Stratified randomization of participants is another strength, which minimizes confounding problems in this randomized controlled trial (RCT). In addition, we will examine the effect of a low-FODMAP diet on different clinical symptoms in patients with IBD. Another strength of the current study is the acquisition of a set of data from participants during the run-in period. Moreover, we will obtain data from participants about dietary intakes and physical activity during the intervention. Finally, along with serum cytokine levels, fecal calprotectin is an important clinically relevant indicator of intestinal inflammation.

### Limitations

The limited number of bacterial species and inflammatory cytokines that will be studied is the greatest limitation of the current project. Although validated questionnaires will be used for assessment of dietary intake and to obtain general information from participants, misclassification of participants cannot be excluded. Furthermore, changes in the intestinal microbiota during the collection process is the greatest concern for the investigators. For this purpose, we will collect fecal samples in the laboratory and store them immediately at − 20 C^0^. Moreover, DNA will be extracted from the sample as soon as possible.

Adherence to a low-FODMAP diet might decrease inflammation through changes in bacterial growth and will probably modulate IBD incidence and progression. Further studies investigating the effect of this diet on other bacterial species and inflammatory cytokines are needed to confirm findings from the current project.

## Trial status

Ongoing, protocol version: 1; 2019-03-19: recruitment began on 1 February 2019 and was completed on 30 May 2019.

## Supplementary information

**Additional file 1.** SPIRIT 2013 Checklist: Recommended items to address in a clinical trial protocol and related documents.

## Data Availability

Data sharing is not applicable to this article as no datasets were generated or analyzed during the current study.

## Abbreviations

BMR: Basic metabolic rate
CBC: Cell blood count
ELISA: Enzyme-linked immunosorbent assay
ESR: Erythrocyte sedimentation rate
FODMAPs: Fermentable oligosaccharides, disaccharides, monosaccharides and polyols
GHQ: General Health Questionnaire
hs-CRP: Highly sensitive-C reactive protein
IBD: Inflammatory bowel disease
IBD-DI: IBD Disability Index
IL: Interleukin
SCFA: Short-chain fatty acid
TEF: Thermogenic effect of foods
TNF-α: Tumor necrosis factor-α
UC: Ulcerative colitis
WC: Waist circumference
